# Endless microbes most beautiful and most wonderful

**DOI:** 10.1371/journal.pgen.1010695

**Published:** 2023-04-20

**Authors:** Gregory S. Barsh, Geraldine Butler, Gregory P. Copenhaver, Sean Crosson, Lotte Søgaard-Andersen, Eva H. Stukenbrock

**Affiliations:** 1 HudsonAlpha Institute for Biotechnology, Huntsville, Alabama, United States of America; 2 Department of Genetics, Stanford University School of Medicine, Stanford, California, United States of America; 3 School of Biomolecular and Biomedical Science, Conway Institute, University College Dublin, Dublin, Ireland; 4 Department of Biology and the Integrative Program for Biological and Genome Sciences, University of North Carolina at Chapel Hill, Chapel Hill, North Carolina, United States of America; 5 Department of Microbiology and Molecular Genetics, Michigan State University, East Lansing, Michigan, United States of America; 6 Department of Ecophysiology, Max Planck Institute for Terrestrial Microbiology, Marburg, Germany; 7 Environmental Genomics, Christian-Albrechts University of Kiel, Kiel, Germany; 8 Max Planck Institute for Evolutionary Biology, Plön, Germany

**Fig 1:**
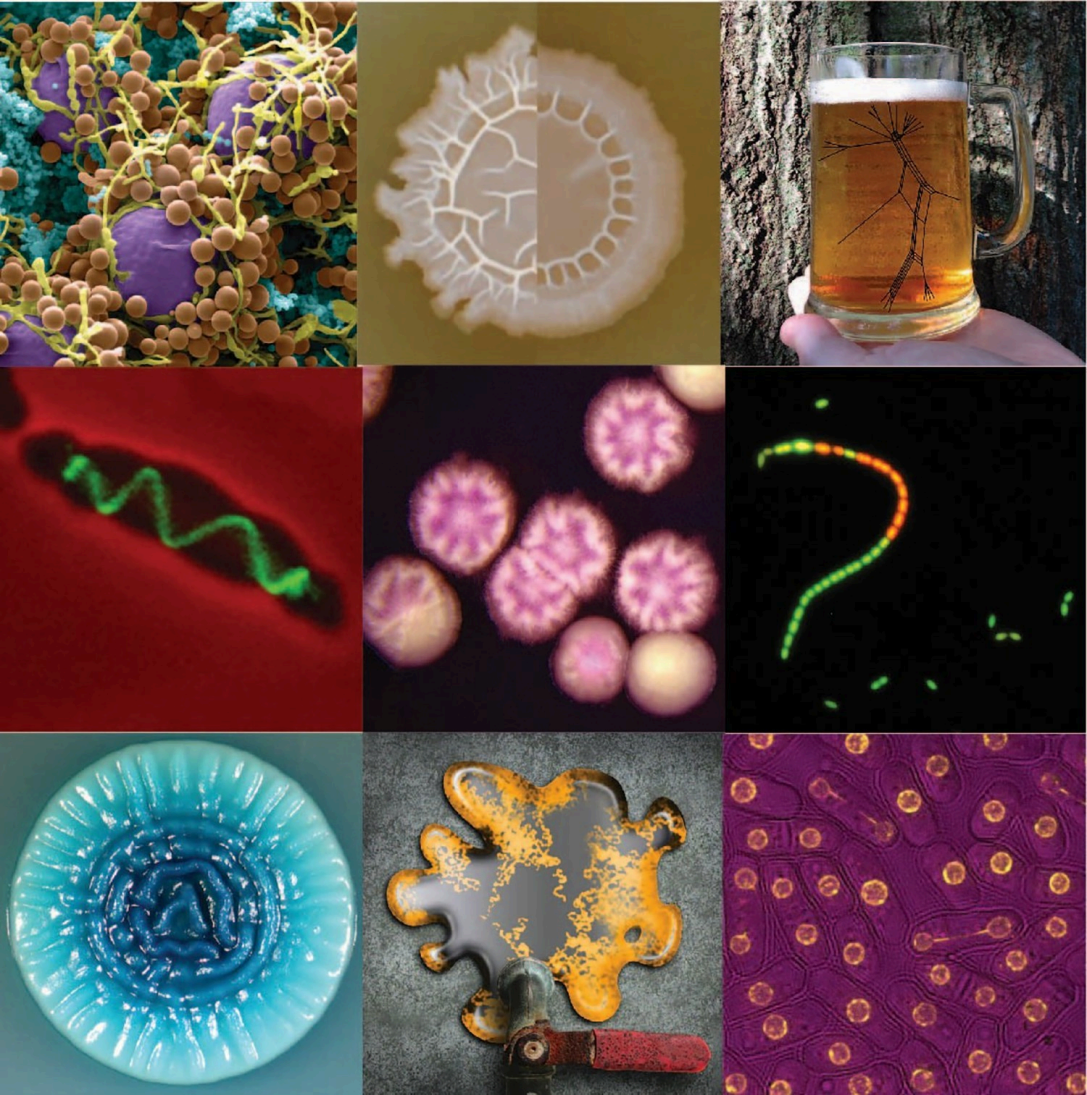
A collage of cover images from previous issues of *PLOS Genetics* showcasing the remarkable and beautiful diversity of the microbial world. Top row: *Aspergillus nidulans* (Image credit: Özgür Bayram and Gerhard H. Braus); *Bacillus subtilis* (Image credit: Kazuo Kobayashi); beer brewed from yeast related to *Saccharomyces eubayanus* (Image credit: A. B. Hulfachor). Middle row: *Streptococcus pneumoniae* (Image credit: Aurore Fleurie); *Candida tropicalis* (Image credit: Han Du, Qiushi Zheng); *Lactococcus lactis* (Image credit: Pascal Le Bourgeois). Bottom row: *Candida albicans* (Image credit: Oliver Homann and Jeanselle Dea); *Rhodococcus opacus* (Image credit: Jason Holder, Leslie Gaffney, Lauren Solomon); *Schizosaccharomyces pombe* (Image credit: Helena Cantwell).

Since Antonie van Leeuwenhoek first observed microbes, we have come to learn that life on our planet includes not only the macroscopic lifeforms visible to the unaided eye that Charles Darwin studied but also an immense diversity of microbes (Fig 1). We have learned that microorganisms influence nearly every aspect of human existence with beneficial or detrimental effects. With their pivotal roles in biomass conversion, biogeochemical cycles, photosynthesis, and in promoting plant growth, life on this planet ultimately depends on the activities of microorganisms. On the other hand, microorganisms are the etiological agents of many diseases in humans, animals, and plants, causing massive economic losses yearly. Microorganisms also contribute significantly to the production of greenhouse gases such as CO_2_ and CH_4_ and, thus, contribute to global warming. In the past decade, we have also learned that microorganisms inhabiting the human body, i.e., the human microbiome, have profound effects on human physiology. Not to forget, some of our most delicious food products and beverages get their distinct qualities from microorganisms, and the pharmaceutical and biotechnological industry relies heavily on microbes. In terms of research, many technological breakthroughs in molecular biology, such as DNA cloning, PCR, and CRISPR-Cas technologies have their origin in microbes. Therefore, research in microbiology is as important now as it ever was.

Research on microbes has developed at a breathtaking pace. Genetic systems have been established in many microbes, and the advent of omics technologies allows studying microbes without established genetic systems. To reflect the importance of microbes and to create a home for microbiology research using genetics, *PLOS Genetics* will be launching a new section on Microbial Genetics. This section will replace the former Prokaryotic Genetics section to emphasize research on microbes more broadly. The new section on Microbial Genetics will cover research on bacteria as well as archaea and their phages/viruses, fungi (including yeasts and filamentous fungi), and protists. We aim to publish studies that use genetic approaches to provide insights of broad significance into how microbes function and interact with the biotic and abiotic world. Manuscripts on genome evolution, comparative genomics, metagenomics, and microbiomes that increase our understanding of microbial diversity will also be included in this new section. Such manuscripts are generally expected to either include functional validation or report a clear genotype–phenotype relationship.

The new section on Microbial Genetics will be headed by Geraldine Butler, Sean Crosson, Eva Stukenbrock, and Lotte Søgaard-Andersen. These four section editors are supported by associate editors with broad expertise in microbial genetics (https://journals.plos.org/plosgenetics/s/editorial-board#loc-associate-editors).

While changing the name of one of our sections may, like a microbe, appear to be a small thing, we hope that its influence will be large. By making our embrace of bacterial, fungal, archaeal, and protists genetics clearer, we look forward to engaging you as readers, reviewers, and authors.

